# A proposed tailored investigational algorithm for women treated for gynaecological cancer with long-term gastrointestinal consequences

**DOI:** 10.1007/s00520-020-05309-z

**Published:** 2020-01-29

**Authors:** Ann Muls, Alexandra Taylor, Susan Lalondrelle, Mohammed Kabir, Christine Norton, Ailsa Hart, H. Jervoise Andreyev

**Affiliations:** 1 The Royal Marsden NHS Foundation Trust, 203 Fulham Road, London, SW3 6JJ UK; 2 Department of Nursing, King’s College London, Waterloo, London, UK; 3 Faculty of Medicine, department of Metabolism, Digestion and Reproduction, Imperial College London, London, UK; 4 The Department of Gastroenterology, Lincoln County Hospital, Lincoln and The School of Medicine, University of Nottingham, Nottingham, UK

**Keywords:** Cancer, Gynaecological malignancy, Chemotherapy, Radiotherapy, Gastrointestinal, Side effects, Toxicity, Quality of life, Bile acid malabsorption, Small intestinal bacterial overgrowth, Endoscopy, Diarrhoea, Abdominal pain, Bloating, Incontinence, Urgency, Late effects, Consequences of treatment

## Abstract

**Background and aim:**

Long-term changes in gastrointestinal function impacting quality of life after treatment for cancer are common. Peer reviewed guidance to investigate and manage GI dysfunction following cancer treatment has been published. This study reviewed gastrointestinal symptoms of women previously treated for gynaecological cancer and considered whether suggested algorithms could be amended to optimise management for this cohort.

**Methods:**

Demographic and clinical data recorded for patients attending a specialist consequences of cancer treatment gastroenterology service prospectively are reported using median and range. The Wilcoxon signed rank test analysed changes in symptoms between initial assessment to discharge from the service.

**Results:**

Between April 2013 and March 2016, 220 women, with a median age of 57 years (range 24–83 years), treated for gynaecological cancer (cervical (50%)), endometrial (28%), ovarian (15%), vaginal or vulval (7%) attended. Twelve gastrointestinal symptoms were statistically significantly reduced by time of discharge from the specialist gastroenterology clinic including bowel frequency ≥ 4/day (88%), type 6 or 7 stool consistency (36%), urgency (31%) and incontinence (21%). General quality of life improved from a median score of 4 at first assessment to a median of 6 at discharge (*p* < 0.001). A median of four (range, 1–9) diagnoses were made.

**Conclusion:**

Women with gastrointestinal symptoms after cancer treatment benefit from a systematic management approach. After excluding disease recurrence, a proposed investigational algorithm and the oncology team includes FBC, U&Es, LFTs, thyroid function test, vitamin B_12_, vitamin D, a hydrogen methane breath test and a SeHCAT scan. If rectal bleeding is present, iron studies, flexible sigmoidoscopy or colonoscopy should be performed. Patients with normal investigations or symptoms not responding to treatment require gastroenterology input.

## Background

Cancer survival has doubled in the last 40 years. Half of people diagnosed with cancer in England and Wales survive for 10 years or more [1]. Advances in multi-modal treatments offer better chances of survival but impact on the body in multiple ways, not only during treatment but long-term. The long-term impact of cancer treatment was the focus of the National Survivorship Initiative and has been adopted in the NHS England cancer plan [2, 3].

The Royal Marsden Gastrointestinal and Nutrition Team (GIANT) service, a clinic established specifically to care for patients experiencing ongoing gastrointestinal symptoms after cancer treatment, is multidisciplinary with input from a consultant gastroenterologist, specialist nurses and dietitians.

The team developed a systematic, checklist-based approach to assess and investigate these long-term gastrointestinal symptoms, tested in the ORBIT study [4]. This showed that patient outcomes when managed by a specialist nurse were not inferior to those managed by a consultant gastroenterologist. Subsequent evaluation of this clinical service suggested that the cost of managing gastrointestinal consequences of cancer treatment across tumour groups averages £1563 per patient [5].

The algorithm used to manage patients’ abnormal symptoms was developed through 10 peer-reviewed versions [6] without regard to the primary cancer site treated. Since different treatment pathways are used for each type of cancer—including type of surgery, chemotherapy regimens, radiotherapy volumes and doses—the predominant causes for long-term gastrointestinal symptoms may vary. This may allow for a tailored algorithm in some patient groups.

Annually, 21,000 UK women are newly diagnosed with a gynaecological malignancy [7]. Following treatment for gynaecological cancer, data are inadequate to assess how many women experience permanent changes to gastrointestinal function which impact on quality of life, activity levels or return to work; however, several studies have highlighted this as a serious concern [8–11].

The aims of this study were to evaluate the symptoms of women previously treated for gynaecological cancer, assessed in a specialist gastroenterology clinic and to review whether the established algorithm could be amended to optimise management in this cohort by the gynaecology team. The study hypothesis was that there would be an improvement in symptom severity at discharge from the gastroenterology clinic compared with baseline.

## Methods

This was an observational study with data collected prospectively on all users of the GIANT service. Following institutional review board approval for service evaluation (SE36), demographic and clinical data were collected on a clinical research form and entered onto database. Data included all requested investigations, resulting gastrointestinal diagnoses and treatments as dictated by the algorithm for patients who completed their episode of care from the clinic.

At each clinic visit, patients self-reported the presence and severity of their symptoms using a modified Gastrointestinal Symptom Rating Scale (GSRS). Symptom severity is categorised as ‘causes major changes in your life’, ‘frequently affecting your life’, ‘occasional’ or ‘never’. The first two categories indicate severe and moderate symptoms. The GSRS has been validated for use in a wide variety of gastrointestinal disorders [12–15]. Patients indicated the frequency of bowel movements and stool consistency using a Bristol stool chart with 1 = hard stool and 7 = liquid stool [16]. In addition, they scored their perceived quality of life (QoL) on a visual analogue scale (VAS) with 0 indicating the worst possible QoL and 10 equating the best possible QoL and the impact of their gastrointestinal symptoms on QoL with 0 indicating no impact and 10 the worst impact possible.

A holistic needs assessment (HNA) was also offered to all patients at first assessment. The HNA comprises a questionnaire covering areas which may concern anyone living with and beyond cancer [2]. Practical, emotional and spiritual family issues can be identified to discuss with a qualified health care professional or highlighted as a concern without discussion, alongside physical concerns. As part of the assessment, the distress thermometer —a screening tool for assessing psychological distress in people affected by cancer—is a requirement of the National Institute of Clinical Excellence guidelines for supportive and palliative care [17]. Patients scored on a scale from 0 (‘I am not distressed’) to 10 (‘I am extremely distressed’). A score of more than 7 justifies a psychological support service referral in a tertiary cancer centre [18].

The Wilcoxon signed rank test was used to analyse changes in symptom burden between initial assessment and the point of discharge from the service. This statistical analysis test allows for comparison of ordinal data (symptom severity) between 2 related groups.

## Results

Between April 2013 and March 2016, there were 1158 new patients and 2686 follow-up appointments in the GIANT clinic. Two hundred thirty-five women (21%) had received treatment for gynaecological malignancy. Fifteen women who declined investigations and further management were excluded, leaving 220 for analysis. Referral to the clinic came from within the organisation (*n* = 94; 43%), other hospitals (*n* = 77; 35%), general practitioners (*n* = 48; 22%) and self-referral (*n* = 1; < 1%).

The primary cancer site was cervix (*n* = 109; 50%), endometrium (*n* = 62; 28%), ovary (*n* = 33; 15%) and vagina or vulva (*n* = 16; 7%). The median age was 57 years (range, 24–83). Several women received multi-modal therapies for their malignancy (Table 1). Nearly 70% (*n* = 151) received radiotherapy (including brachytherapy), 54% (*n* = 118) had surgery and 45% (*n* = 98) chemotherapy. Seventeen percent (*n* = 37) received all three therapies. Treatment characteristics per tumour group are presented in Table 1. The median time between cancer diagnosis and referral was 4 years and 10 months (range, 6 months–47.5 years). Ten percent of women had been seen previously and were re-referred. In patients seen for the first time, the median number of consultations was 4 (range, 1–17) with 38% requiring more than four. Those who were re-referred had a median of 4 consultations (range, 1–7) with 30% needing more than four.

**Table 1:** Cancer treatment regimens per cancer diagnosis group (*n* = 220)

	Cervical cancer, *n* (%)	Endometrial cancer, *n* (%)	Ovarian cancer, *n* (%)	Vaginal/vulval cancer, *n* (%)	Total, *n* (%)
Cancer treatment					
Chemotherapy alone	11	1	8	2	22 (10%)
Radiotherapy alone	36	13	1	2	52 (24%)
Surgery alone	10	16	11	2	39 (18%)
CXT + RT (no surgery)	24	1	0	3	28 (13%)
CXT + surgery (no RT)	0	0	8	3	11 (5%)
RT + surgery (no CXT)	12	18	0	1	31 (14%)
CXT + RT + surgery	16	13	5	3	37 (17%)
Total	109 (50%)	62 (28%)	33 (15%)	16 (7%)	220 (100%)
Additional cancer treatment information per cancer diagnosis group (*n* = 220)					
EBRT w/o brachytherapy	37	29	5	4	71 (32%)
Brachytherapy w/o EBRT	3	3	0	1	7 (3%)
EBRT + brachytherapy	48	13	4	4	69 (31%)
RT total	88 (40%)	45 (20%)	9 (4%)	9 (4%)	151 (69%)
Chemotherapy total	51 (23%)	15 (7%)	21 (10)	11 (5%)	98 (45%)
Surgery total	38 (17%)	47 (21%)	24 (11%)	9 (4%)	118 (54%)
Hormone replacement therapy	1	4	4	0	9 (4%)
Other treatment	0	0	0	0	0
Colostomy	1	0	0	1	2 (1%)

**Fig. 1:**
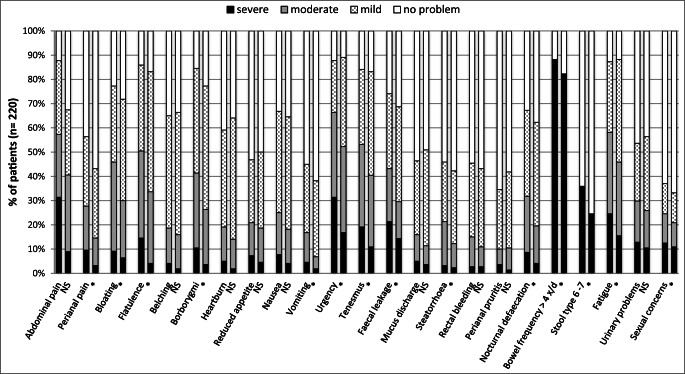
Paired symptom scores (*n* = 220) at baseline and discharge; left bar, baseline assessment; right bar, discharge assessment (NS, not significant, *p < 0.05)

The most prevalent gastrointestinal symptoms at assessment rated as severe included increased frequency of defaecation (88%), diarrhoea (36%), urgency (31%) and incontinence (21%). In addition, many women also reported fatigue (87%), urinary problems (53%) and sexual concerns (38%) (Fig. 1).

A median of 8 (range, 1–14) investigations were requested for this cohort using our algorithm version 10 [6]. These included blood screen (*n* = 213; 97%), gastroscopy (*n* = 178; 80%) with duodenal aspirate (*n* = 168; 76%), glucose hydrogen methane breath test (*n* = 177; 80%), SeHCAT scan (*n* = 161; 73%), stool sample for faecal elastase (*n* = 152; 69%), flexible sigmoidoscopy (*n* = 114; 52%), colonoscopy (*n* = 56; 25%) and abdominal X-ray (*n* = 35; 16%).

These investigations revealed a median of four abnormal findings (range, 1–9) with 68% of women (*n* = 150) having more than three. Blood screens showed that vitamin D deficiency is common in this group (60%). The vitamin B_12_ test was below the normal range for 30% of this cohort (*n* = 65). Thyroid function tests indicated abnormal values for 5% (*n* = 11) (Table 2).

**Table 2:** Prevalence of new gastrointestinal or nutritional diagnoses (*n* = 220)

Diagnosis	Prevalence, *n* (%)
Vitamin D deficiency	133 (60%)
Small intestinal bacterial overgrowth	118 (54%)
Bile acid malabsorption	104 (47%)
Gastritis	68 (31%)
Vitamin B_12_ deficiency	65 (30%)
Weak pelvic floor musculature on rectal exam	36 (21%)
Telangiectasia on the rectal wall	33 (15%)
Trace element deficiency	31 (14%)
New GI polyp	24 (11%)
Hiatus hernia	22 (10%)
Faecal loading (confirmed on abdominal X-ray)	18 (8%)
Dietary fibre excess on fibre quiz	16 (7%)
Oesophagitis	16 (7%)
Iron deficiency	16 (7%)
Pancreatic insufficiency	16 (7%)
Duodenitis	13 (6%)
Thyroid problems	11 (5%)
Diverticular disease	9 (4%)
Gastro-oesophageal reflux disease	8 (4%)
Haemorrhoids	7 (3%)
Inflammatory bowel disease	4 (2%)
Rectal ulcer	4 (2%)
New GI cancer	5 (2%)
Anal fissure/anal sphincter defect	3 (1%)

Following the algorithm, in the presence of upper GI symptoms, a gastroscopy with duodenal aspirate was often ordered to test for small intestinal bacterial overgrowth (SIBO) in addition to upper gastrointestinal pathology. In this cohort, more than 75% (*n* = 168) of women had a duodenal aspirate. Of those, 60% (*n* = 101) did not report any growth. Of the 40% (*n* = 67) that did, half of the cases (*n* = 35) had sensitivities reported resulting in specific antibiotic treatment which in 15% (*n* = 5) differed from the recommendation of the algorithm. One hundred seventy-seven women had a glucose hydrogen methane breath test which was positive for both hydrogen and methane in 24% (*n* = 43), for methane alone in 12% (*n* = 22) and for hydrogen alone in 8% (*n* = 14). Nearly a third of women (*n* = 33) with persistent symptoms required multiple treatments with antibiotics (range, 2–9) and two women required long-term, rotating antibiotics.

Bile acid malabsorption was a common diagnosis in this group of women (*n* = 104; 47%); 73% (*n* = 161) had a SeHCAT scan which was positive in 65% (*n* = 104). The algorithm suggests that a SeHCAT should be requested for anyone reporting type 6 or 7 stool consistency, urgency or steatorrhoea [6]. For those women who reported urgency without diarrhoea, 60% (*n* = 54) had a positive SeHCAT scan.

Faecal loading complicated by overflow diarrhoea was diagnosed following an abdominal X-ray in 8% of the cohort (*n* = 18).

A stool sample for faecal elastase, sent in 69% (*n* = 152), confirmed pancreatic insufficiency in 7% (*n* = 16) requiring treatment with enzyme replacement and sometimes specialist dietetic advice. A coeliac screen was requested in 84% of the women (*n* = 185) but did not identify any women with the condition.

**Fig. 2:**
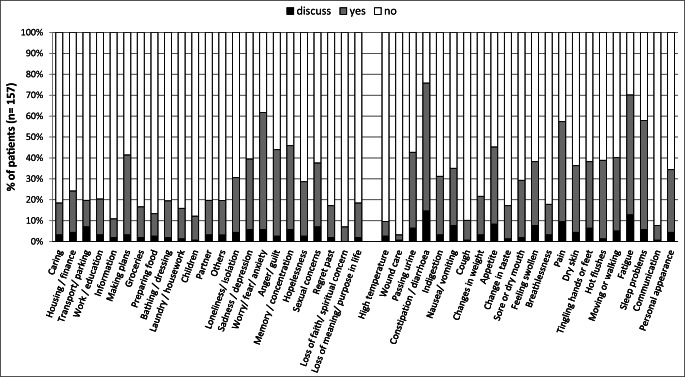
Concerns highlighted by women treated for gynaecological malignancy on the holistic needs assessment (*n* = 157)

The holistic needs assessment was completed by 71% (*n* = 157) (Fig. 2). The main concerns for women after treatment for gynaecological cancer were fatigue (70%), worry, fear and anxiety (62%), sleeping problems (58%) and pain (57%). Women scored a median of 5 (range, 0–10) on the distress thermometer and 35% (*n* = 59) scored 7 or more.

Twelve gastrointestinal symptoms were statistically significantly reduced by time of discharge from the specialist gastroenterology clinic (Table 3) (*p* < 0.05). The general quality of life score improved from a median of 4 at first assessment to a median of 6 at discharge (*p* < 0.001). The impact of residual or recurrent gastrointestinal symptoms on QoL did not improve with a median of 7 out of a maximum score of 10 (*p* > 0.05).

**Table 3:** Symptoms which improved at a statistically significant level (*p* < 0.05), *n* = 220

Symptoms	Baseline (*n* = 220)	Discharge (*n* = 220)	*p* value
	*n* (%)	*n* (%)	
Perianal pain			
No problem	96 (44)	125 (57)	
Mild	63 (29)	63 (29)	< 0.001
Moderate	40 (18)	25 (11)	
Severe	21 (10)	7 (3)	
Bloating			
No problem	50 (23)	62 (28)	
Mild	69 (31)	92 (42)	< 0.001
Moderate	81 (37)	52 (24)	
Severe	20 (9)	14 (6)	
Flatulence			
No problem	31 (14)	37 (17)	
Mild	78 (35)	109(50)	< 0.001
Moderate	79 (36)	65 (30)	
Severe	32 (15)	9 (4)	
Borborygmi			
No problem	34 (15)	50 (23)	
Mild	95 (43)	112 (51)	< 0.001
Moderate	68 (31)	50 (23)	
Severe	23 (10)	8 (4)	
Vomiting			
No problem	121 (55)	136 (62)	
Mild	62 (28)	69 (31)	0.002
Moderate	27 (12)	11 (5)	
Severe	10 (5)	4(2)	
Urgency			
No problem	27 (12)	24 (11)	
Mild	47 (21)	81 (37)	< 0.001
Moderate	77 (35)	78 (35)	
Severe	69 (31)	37 (17)	
Tenesmus			
No problem	35 (16)	37 (17)	0.003
Mild	68 (31)	94 (43)	
Moderate	75 (34)	65 (30)	
Severe	42 (19)	24 (10)	
Faecal incontinence			
No problem	50 (23)	60 (28)	
Mild	59 (27)	85 (39)	< 0.001
Moderate	58 (26)	45 (20)	
Severe	53 (24)	30 (14)	
Steatorrhoea			
No problem	119 (54)	127 (58)	
Mild	54 (25)	66 (30)	0.045
Moderate	40 (18)	22 (10)	
Severe	7 (3)	5 (2)	
Nocturnal defaecation			
No problem	72 (33)	83 (38)	
Mild	78 (35)	94 (43)	< 0.001
Moderate	51 (23)	34 (15)	
Severe	19 (9)	9 (4)	
Bowel frequency > 4x/day			
No	26 (12)	39 (18)	0.009
Yes	194 (88)	181 (82)	
Diarrhoea (type 6 or 7)			
No	142 (65)	165 (75)	0.013
Yes	78 (35)	55 (25)	
Fatigue			
No problem	28 (13)	26 (12)	
Mild	64 (29)	93 (42)	0.001
Moderate	74 (34)	67 (30)	
Severe	54 (26)	34 (15)	
Sexual concerns			
No problem	137 (62)	147 (67)	
Mild	28 (13)	27 (12)	0.004
Moderate	27 (12)	22 (10)	
Severe	28 (13)	24 (11)	

## Discussion

This prospective cohort study shows that a specialist gastroenterology service can improve the outcomes for women living with and beyond cancer after treatment for gynaecological malignancy by reducing gastrointestinal symptom burden and improving quality of life.

The findings of this study are consistent with other studies [19] which show that the main concerns for women on holistic needs assessment are largely unchanged from their concerns at the end of treatment and in 1 in 3 justify a psychological support service referral because of worry, fear and anxiety, fatigue and sleeping problems.

The relatively high prevalence of symptoms still present at discharge may partly be related to the fact that whilst specific treatments help improve symptoms, as yet there are no proven treatments to tackle the underlying pathological changes which cause pelvic radiation disease [20]. Despite this, there remains scope for further improvement. Support delivered by a specialist multidisciplinary team may have a beneficial psychological effect in itself. Furthermore, the importance to provide person-centred care and use patient recorded outcome measures (PROMS) is highlighted by the suggestion that even though general QoL improves from baseline, the impact of residual or recurring gastrointestinal symptoms on QoL remains the same at discharge. This may be due to heightened awareness of the causes contributing to these symptoms and need for ongoing, long-term management strategies including demanding lifestyle and dietary changes.

Whilst the reduction in severity reached statistical significance in 12 out of 20 gastrointestinal symptoms, the average time from diagnosis of cancer to referral to a specialist gastroenterology service was nearly 5 years. There is a need for earlier identification of patients requiring intervention. One way to do this is by developing assessment measures such as the ALERT-B questionnaire validated for use in oncology follow-up clinics in patients with prostate cancer who may benefit from further assessment [21]. After excluding disease progression, the next logical step would be for the oncology team to initiate first-line investigations to identify treatable symptoms. To this end, this study suggests an investigational algorithm tailored to symptomatic gynaecological oncology patients which any team should not find challenging. If symptoms remain unresolved or these tests are negative, a referral can be made to specialist gastroenterology services (Table 4).

**Table 4:** Investigation algorithm tailored for women treated for gynaecological cancer with ongoing GI problems

Investigational algorithm tailored for gynaecological cancer	Original investigational algorithm (Frontline Gastroenterology, 2015)
**First-line investigations (oncology clinic)**	**First-line investigations**
Blood screen: FBC, U&Es, LFTs Vitamin D Vitamin B_12_	Blood screen: FBC, U&Es, LFTs, CRP, ESR Vitamin D Vitamin B_12_ Iron studies
Glucose hydrogen methane breath	
SeHCAT scan	Thyroid function Coeliac screen Serum magnesium
If rectal bleeding is present: iron studies Flexible sigmoidoscopy or colonoscopy (avoid biopsies of irradiated tissue)	
If no rectal bleeding but anaemic: iron studies Flexible sigmoidoscopy or colonoscopy (avoid biopsies of irradiated tissue)	Stool sample for microscopy, culture and *Clostridium difficile*
	Stool sample for faecal elastase
**Second-line investigations (gastroenterology team)**	Glucose hydrogen methane breath test Gastroscopy with duodenal aspirate
Stool sample for faecal elastase	Carbohydrate challenge
Coeliac screen	SeHCAT scan
Thyroid function screen	Flexible sigmoidoscopy/colonoscopy (avoid biopsies of irradiated tissue)
Abdominal X-ray	**Second-line investigations**
**Third-line investigations (gastroenterology team)**	Colonoscopy with biopsies
Carbohydrate challenge	**Third-line investigations**
Gut hormone blood test, urinary 5-HIAA	Gut hormone challenge
Colonoscopy with biopsies	Urinary 5-HIAA
CT chest abdomen and pelvis/CT colonography to exclude structural GI pathology	CT chest abdomen and pelvis

Within this cohort, several tumour sub-groups were represented with different treatment regimens. Whilst nearly 70% received radiotherapy to the pelvic area, nearly 50% also received other treatments, making it difficult to establish which treatment modality contributes to gastrointestinal symptom burden. The heterogeneity of the patients included in the study regarding tumour and treatment types could limit the applicability of the algorithm to each different cancer type, treatment and stage. Further testing is needed to provide evidence of its use in clinical practice and its merit in reducing symptom burden for these patients.

In this study, two-thirds of women were deficient in vitamin D. Clinical evidence supports that adequate vitamin D levels or supplementation in those with low levels reduces the risk of developing gynaecological malignancies although the molecular pathways remain poorly understood [22]. Vitamin D is essential to aide the absorption of calcium and maintain bone health [23] and after pelvic radiotherapy patients are at increased risk of bone fractures. Whilst in the general population supplementation of vitamin D does not improve bone health [24], this may be different in a cancer population and people with bile acid malabsorption.

Vitamin B_12_ deficiency is a potent cause for tiredness and is linked to memory problems, anaemia and breathlessness. Levels are often low in those with bile acid malabsorption and SIBO [25–27]. Up to 23% of women treated for gynaecological cancer were previously reported to have decreased vitamin B_12_ levels [28] and in this cohort, it was detected in 30%.

Interpretation of all currently available investigations to diagnose SIBO is problematic [29] and it is difficult to ascertain how effective treatment for SIBO is without systematic re-testing. A breath test should remain a first-line test in this patient cohort. In view of antibiotic stewardship [30], a duodenal aspirate may be helpful in people with recurrent SIBO or persistent symptoms.

Bile acid diarrhoea has an estimated prevalence in the general population of 1% and is frequently misdiagnosed as IBS [31, 32]. Bile acid malabsorption was diagnosed in 47% in our cohort. Some of these may have had primary bile acid diarrhoea and unrecognized pre-treatment, which exacerbated during treatment but for many, new onset symptoms were likely secondary to the effect of chemo-radiotherapy on the absorptive capacity for bile of the terminal ileum. Our finding is in keeping with other studies. Bile acid malabsorption is often thought as causing chronic watery stool. We have previously shown that a wide spectrum of symptoms improves when bile acid malabsorption is adequately treated [33]. It is therefore particularly interesting that 40% of patients in whom we made this diagnosis only had urgency and did not report loose stool. In some, opioids or anti-diarrhoeal medication may have masked their diarrhoea; this highlights that urgency in this patient group may be a marker for bile acid malabsorption.

Constipation is common in up to 60% of cancer patients and can be due to several causes following treatment: small intestinal bacterial overgrowth, reduced dietary fibre intake, medication, anorectal pain, anal fissure and co-morbidities such as diabetes [34]. Although bowel obstruction occurs more often in women treated for ovarian cancer, it is important to differentiate this from severe faecal loading through imaging [35]. In this cohort, abdominal X-ray had a 50% yield for identifying faecal loading which was thought to predispose to abdominal pain or overflow diarrhoea.

In this group, a stool sample for faecal elastase, which costs about £39, has a 7% yield for diagnosing pancreatic insufficiency. Thyroid function problems were detected in only 5% of patients but are routinely included in our blood screen which was done in 97% of the cohort. A coeliac screen is also routinely included, especially as people often cut gluten out of their diet. The cost of a coeliac screen is about 25–£40 with on-costs (personal communication). As we did not identify any new onset of coeliac disease in this cohort, and the low yield of faecal elastase and coeliac screening, it may be reasonable not to include them in the oncology clinic; however, this may delay diagnosis, affect clinic capacity and potentially increase costs as patients may require additional appointments before treatment has been optimised.

The most common and severe symptoms in this cohort were urgency and type 6 or 7 stool consistency. A new tiered approach to investigation (Table 2) in this cohort may facilitate the management of symptoms as it can be difficult to know which intervention has resulted in improvement or failed to have an impact.

In this prospective cohort, we were unable to correlate gastrointestinal symptoms to disease stage, treatment modalities, radiotherapy volumes or doses but this is important to include in further studies. Differences in disease stage could have influenced symptom incidence, severity and outcome. Pelvic radiotherapy is used to treat gynaecological malignancies in 30% of women. Moderate to severe long-term gastrointestinal symptoms are reported by clinicians in 5–15% of patients treated [36] and up to 50% when PROMs are used [37, 38]. Extrapolating the number of patients treated for gynaecological malignancies with pelvic radiotherapy, this would mean up to 3225 women need access to this kind of service in the UK where 21,500 gynaecological cancer diagnoses are made yearly [7].

## Conclusion

Significant improvement in gastrointestinal symptoms for women with long-term bowel problems following treatment for gynaecological cancer can be achieved. The most common functional deficits are bile acid malabsorption, small intestinal bacterial overgrowth and vitamin D deficiency. The proposed investigational algorithm tailored to this cohort will need to be tested further in clinical practice to ascertain its clinical value. There is a need for earlier intervention and further research for predicting and treating bowel toxicity which correlates GI symptom burden with radiotherapy volumes and doses.
